# Natural disease history and characterisation of *SUMF1* molecular defects in ten unrelated patients with multiple sulfatase deficiency

**DOI:** 10.1186/s13023-015-0244-7

**Published:** 2015-03-15

**Authors:** Frédérique Sabourdy, Lionel Mourey, Emmanuelle Le Trionnaire, Nathalie Bednarek, Catherine Caillaud, Yves Chaix, Marie-Ange Delrue, Anne Dusser, Roseline Froissart, Roselyne Garnotel, Nathalie Guffon, André Megarbane, Hélène Ogier de Baulny, Jean-Michel Pédespan, Samia Pichard, Vassili Valayannopoulos, Alain Verloes, Thierry Levade

**Affiliations:** Laboratoire de Biochimie Métabolique, IFB, CHU Purpan, Toulouse, France; INSERM UMR 1037, CRCT, Université Paul Sabatier Toulouse-III, Toulouse, France; Institut de Pharmacologie et de Biologie Structurale (IPBS), Centre National de la Recherche Scientifique (CNRS), Toulouse, France; Université de Toulouse, Université Paul Sabatier, IPBS, Toulouse, France; Service de Néonatologie, Alix de Champagne, CHU de Reims, Reims, France; Laboratoire de Biochimie, Métabolomique et Protéomique, Hôpital Necker Enfants Malades, Paris, France; Hôpital des Enfants, CHU Purpan, and INSERM UMR 825 Imagerie Cérébrale et Handicaps Neurologiques, Université Paul Sabatier Toulouse-III, CHU Purpan, Toulouse, France; Service de Génétique Médicale, CHU Pellegrin, et laboratoire Maladies Rares, Génétique et Métabolisme, Université Bordeaux 2, Bordeaux, France; Service de Neuropédiatrie, CHU de Bicêtre, Kremlin-Bicêtre, France; Centre de Biologie et Pathologie Est, Hospices Civils de Lyon, Bron, France; Laboratoire de biologie et de recherche pédiatriques, American Memorial Hospital, CHU Reims, Reims, France; Centre de Référence des Maladies Héréditaires du Métabolisme, Lyon, France; Unité de Génétique Médicale et Laboratoire Associé INSERM UMR_S910, Université Saint-Joseph, Beirut, Lebanon; Institut Jérôme Lejeune, Paris, France; Centre Référence des Maladies Héréditaires du Métabolisme, CHU Robert Debré, APHP, Paris, France; Service de neurologie pédiatrique, Université Bordeaux 2, Bordeaux, France; Centre de référence-maladies métaboliques, Hôpital Necker Enfants malades, Paris, France; Service de Génétique Clinique, CHU Robert Debré, Paris, France

**Keywords:** Multiple sulfatase deficiency, Mucosulfatidosis, *SUMF1*, FGE, Genotype-phenotype correlations

## Abstract

**Background:**

Multiple sulfatase deficiency is a rare inherited metabolic disorder caused by mutations in the *SUMF1* gene. The disease remains poorly known, often leading to a late diagnosis. This study aimed to provide improved knowledge of the disease, through complete clinical, biochemical, and molecular descriptions of a cohort of unrelated patients. The main objective was to identify prognostic markers, both phenotypic and genotypic, to accelerate the diagnosis and improve patient care.

**Methods:**

The phenotypes of ten unrelated patients were fully documented at the clinical and biochemical levels. The long-term follow-up of each patient allowed correlations of the phenotypes to the disease outcomes. Each patient’s molecular defects were also identified. Site-directed mutagenesis was used to individually express the mutants and assess their stability. Characterisation of the protein mutants was completed by *in silico* analyses based on sequence comparisons and structural models.

**Results:**

The most severe cases were characterised by the presence of non-neurological symptoms as well as the occurrence of psychomotor regression before 2 years of age. Nine novel *SUMF1* mutations were identified. Clinically severe forms were often associated with *SUMF1* mutations that strongly affected the protein stability and/or catalytic function as predicted from *in silico* and western blot analyses.

**Conclusions:**

This detailed clinical description and follow-up of a cohort of patients, together with the molecular characterisation of their underlying defects, contribute to improved knowledge of multiple sulfatase deficiency. Predictors of a bad prognosis were the presence of several non-neurological symptoms and the onset of psychomotor regression before 2 years of age. No strict correlation existed between in vitro residual sulfatase activity and disease severity. Genotype–phenotype correlations related to previously reported mutants were strengthened. These and previous observations allow not only improved prediction of the disease outcome but also provision of appropriate care for patients, in the expectation of specific treatment development.

**Electronic supplementary material:**

The online version of this article (doi:10.1186/s13023-015-0244-7) contains supplementary material, which is available to authorized users.

## Background

Multiple sulfatase deficiency (MSD; MIM #272200), also known as Austin disease or mucosulfatidosis, is a very rare autosomal recessive disorder. It was first described in 1964 by Austin as an atypical form of metachromatic leukodystrophy (MLD), a disease caused by deficient arylsulfatase A activity [1,2]. In addition to the neurodegenerative syndrome typically observed in MLD, the patients described by Austin presented with symptoms reminiscent of other sulfatase deficiencies, such as mucopolysaccharidoses (MPS) and X-linked ichthyosis. Biochemical analyses revealed that the activities of several sulfatases were indeed simultaneously decreased in the cells of these patients. MSD is now clearly defined as a single genetic entity characterised by the deficient activity of all cellular sulfatases, regardless of their subcellular localisation [3,4]. In fact, all 17 currently known sulfatases, including lysosomal, microsomal, Golgi, and cell surface sulfatases, are concomitantly affected. Patients with MSD exhibit combined symptoms from several individual sulfatase deficiency disorders, leading to heterogeneous clinical presentations. Based on the main symptoms and ages of their onset, four clinical subtypes of MSD with varying severity have been distinguished [5]. The neonatal subtype is characterised by MPS-like symptoms occurring in the first months of life, and usually leads to early death before 1 year of age. The late infantile forms are rather dominated by progressive neurodegeneration, such as that observed in MLD. The distinction between the severe and mild late infantile subtypes is based on the age of onset of symptoms (i.e., before or after 2 years of age), as well as the number of typical signs of individual sulfatase deficiencies. The juvenile subtype is characterised by a late onset and attenuated symptoms.

The issue of how a single gene defect can affect a whole enzyme family remained unsolved for four decades, until the molecular basis of MSD was simultaneously discovered by two groups [6,7]. Mutations in the *Sulfatase Modifying Factor 1* (*SUMF1*) gene (chromosome 3p26.1) were found to lead to a functional defect in formylglycine-generating enzyme (FGE), a critical protein involved in the post-translational activation of all sulfatases in the endoplasmic reticulum. FGE oxidises a cysteine residue located within a consensus CxPxR sequence shared by all sulfatases to generate a formylglycine. This cysteine oxidation is absolutely required by sulfatases to exert their enzymatic activity.

Despite the recent identification of the molecular bases underlying MSD, the disease is still under-diagnosed and poorly known. Less than 50 patients suffering from this rare disease have been reported to date [8]. Although an animal model of the disease has been generated [9], its pathophysiology remains unclear. Disturbed autophagy has been described [10]. Furthermore, although gene therapy trials have recently been performed on animal models [11], no specific therapy is yet available for this very severe disorder. Improved knowledge regarding the molecular bases of MSD is thus needed.

Since the discovery of FGE, significant advances have been made toward the clarification of its molecular and biochemical properties. Determination of the three-dimensional structure of the protein has contributed to the identification of several critical residues involved in FGE stability and catalytic activity [12-14]. In addition, analyses of the *SUMF1* mutations found in MSD patients have proven to be very informative. Specifically, about 30 different mutations have been reported to date. Some of these mutant genes were individually expressed to assess their impact on FGE expression and catalytic activity [6,7,12,15-17]. Taken together, the resulting data suggested an original enzymatic mechanism for the sulfatase cysteine oxidation to formylglycine. Previous studies have also allowed some conclusions to be drawn regarding genotype–phenotype correlations. For example, mutations affecting both the stability and catalytic activity of FGE seem to be responsible for the most severe phenotypes, whereas mutations that exclusively impair one of these properties tend to be associated with milder forms of the disease [18].

To further improve our understanding of MSD, we report here 10 novel unrelated patients whose phenotypes and genotypes were characterised. Based on complete descriptions of the clinical presentation and disease outcome of each patient, as well as the associated biochemical and molecular defects, features that can influence the prognosis of MSD are presented.

## Methods

### Patients

Ten patients from unrelated families in which clinical examination and lysosomal enzyme analyses led to MSD diagnosis were investigated for *SUMF1* mutations. Detailed clinical features and biochemical data are presented in the results section. Signed, informed consent was obtained from each patient and their parents.

### Genetic analyses

Genomic DNA was isolated from peripheral blood leukocytes (Nucleospin II, Macherey-Nagel, Germany). Exons 1 through 9 of the *SUMF1* gene including intron-exon junctions were individually amplified [19] and sequenced in both directions. For cDNA analysis, RNA was isolated from Epstein-Barr virus-transformed lymphoid cells (SV Total RNA extraction kit, Promega), reverse-transcribed and amplified (primer sequences are available upon request). DNA sequencing was performed using an ABI3100 Applied Biosystems automatic sequencer.

### Expression of wild-type and mutant FGE proteins

#### Site-directed mutagenesis of the SUMF1 cDNA

The pSUMF1-3xFlag plasmid was kindly provided by Dr. A. Fraldi (Naples, Italy) [20]. This construct was used as a template for site-directed mutagenesis using the QuickChange Site-Directed Mutagenesis Kit (Stratagene) and primer pairs are listed (Additional file 1). The *SUMF1* cDNA of patient 7 carrying the V174-P318dup duplication was amplified and inserted into p3XFlag-CMV, in frame with the Flag tag. All recombinant vectors were purified (Qiagen Plamid Maxi kit) and the inserts sequenced.

#### Cell transfection

HEK293T cells were grown in a humidified 5% CO_2_ atmosphere at 37°C in DMEM containing Glutamax (2 mM) and 10% heat-inactivated fetal calf serum (FCS). Cells were transfected with 5 μg of either pSUMF1-3xFlag, pR236X-Flag, pN259S-Flag, pG263V-Flag, pA298E-Flag, pY340H-Flag, pR343S-Flag, or pV174-P318dup-Flag plasmids, using Superfect (Qiagen). After 48 h incubation, HEK293T cells were washed with PBS and harvested. Cell pellets were frozen at −80°C until use.

### Western blot analysis

Cell pellets were resuspended in lysis buffer (Cell Signaling) containing 1 mM PMSF and a protease inhibitor cocktail (Complete, Roche). Protein extracts (25 μg) were loaded and separated in a 10% SDS-polyacrylamide gel and transferred to a nitrocellulose membrane (Bio-Rad). FGE was detected by using an anti-Flag mouse monoclonal antibody (1:1000, Sigma). Anti-β-actin and goat anti-mouse secondary antibodies were from Cell Signaling.

### In silico analysis of FGE mutants

The Polyphen software was used to predict the possible impact of amino acid substitutions on the structure and function of the FGE protein [21]. The Human Splicing Finder software [22] allowed to predict potential transcripts induced by mutations occurring at splicing sites. The Swiss-model program [23-25] was used to deduce hypothetical structures from the sequences of FGE mutants. The 1Y1E.pdb and 1Z70.pdb structures were used as templates, and homology models were produced using the Automated mode. Structure visualization and analyses were carried out using the PyMOL program [26].

## Results

### Clinical data

Patient 1 was born to non-consanguineous parents after an uneventful pregnancy. The birth parameters were in the normal range (BW, 3.150 kg; BL, 50.5 cm; HC, 34 cm). The disease was revealed by mild motor delay with hypotonia contrasting with good verbal abilities during the first 3 years of life. Although there was no motor regression, cerebellar ataxia with generalised tremor appeared between 4 and 7 years, followed by nystagmiform movements revealing retinitis pigmentosa. MRI showed a thin corpus callosum and cerebellar atrophy. In the following years, the patient improved his motor abilities and language. No further neurological deterioration, swallowing difficulties, or spasticity were noted. He developed mild ichthyosis that became prominent during the summer period. At the last clinical evaluation, he was 12 years old. He measured 143 cm, and weighed 32.9 kg (normal range). His skin appeared normal. He had no walking difficulties, but experienced tremor of the upper limbs. His major impairment was visual loss.

Patient 2 was born to non-consanguineous parents. He presented with an inguinal hernia at birth (repaired surgically at 16 months) and experienced repeated otitis media in the first year of life. He required tympanostomy and tonsillectomy. Speech and psychomotor delays were noticed around 2 years of age, with delay in toilet training and socialisation difficulties suggesting atypical autism. The disease progressed slowly with minor physical changes: he displayed claw fingers, abnormal ear helix, and microretrognathia. MRI showed demyelination, ventricular enlargement, and cerebral atrophy. In the following years, the patient developed ichthyosis, psychomotor regression, and growth retardation. He was able to communicate using a few words until 7 years of age, but then progressively lost speech. Sleep disturbance was noticed from the age of 8 years, along with signs of retinitis pigmentosa. At his last follow-up at 11 years of age, he presented with MPS-like dysmorphism associated with severe spasticity. His weight was 21.5 kg (< −3 SD) for a height of 117 cm (< −3 SD).

Patient 3 was born to non-consanguineous parents after a pregnancy marked by third trimester foetal distress. She had low birth weight and height (BW, 2.670 kg (−1.5 SD); BL, 46 cm (−2 SD); HC, 34.5 cm). The disease was revealed by markedly delayed motor skills at 12 months and hypotonia at 18 months, along with delayed verbal abilities. She crawled at 18 months and walked unsupported at 2.5 years. Speech regression and loss of communication occurred around 2.5 years of age. She could pronounce a few words at 5 years. Brisk tendon reflexes at the lower limbs and truncular ataxia were noticed at 6 years. Brain MRI showed mild cortical atrophy, and cerebellar atrophy with vermian hypoplasia. She also presented with severe limb vasomotor impairment, mimicking Raynaud’s syndrome. At 7 years of age, the patient was still able to walk without assistance. She was unable to be toilet-trained, but could speak a dozen disyllabic words. Although autism spectrum disorder was not formally assessed, her behaviour showed many autistic features, such as poor visual contact and hand stereotypies, for which applied behaviour analysis therapy was set up. Ophthalmological evaluation revealed oculomotor apraxia and severe myopia. The parents noted progress in manual skills, walking, social interactions, and comprehension. At 9 years of age, clinical examination revealed hepatomegaly, hip laxity, and enhanced ataxia. She lost autonomous walking ability and needed support to climb stairs. At the age of 10 years and 6 months, she measured 124.5 cm (−2.5 SD), weighed 26 kg (−2 SD), and had a HC of 51 cm (−1.5 SD). She had no facial dysmorphism.

Patient 4 was born to non-consanguineous parents after an uneventful pregnancy (BW, 2.740 kg (−1.5 SD); BL, 46 cm (−2 SD)). Her neurological development was mildly delayed: she was able to walk at 18 months, and pronounced her first words between 18 and 24 months of age. From 24 to 36 months of age, her mother reported a slowdown in language acquisition, with fluctuation in speech production. The patient suffered from recurrent serous otitis and infections of the upper respiratory tract that required adenotonsillectomy. She attended school, and started speech therapy and psychomotor rehabilitation. She was hospitalised at the age of 3 years and 5 months because of psychomotor delay and major feeding difficulties. Unsupported walking was possible, but complicated by frequent falls. She was able to climb stairs with assistance. Speech was delayed and she could only pronounce 10 isolated words. Neurological examination revealed ataxia, brisk tendon reflexes, and hypotonia of the upper and lower limbs. Microcephaly was noted (HC: 47 cm (−2 SD)). Audition was assessed by auditory-evoked responses, revealing mixed hearing loss. Brain MRI performed at 4 years and 8 months showed cerebellar hypoplasia. Taken together, all of these findings pointed to a progressive neurological disease, which was finally diagnosed as MSD. At the last evaluation, she was 6 years and 2 months old, still able to walk 50 meters alone but with difficulties due to a marked ataxia. She could pronounce only a few disyllabic words but interacted well with her environment. She was agitated and suffered from sleep disturbances.

Patient 5 was born to non-consanguineous parents after a normal pregnancy (BW, 3.990 kg; BL, 51 cm; HC, 34 cm (−1 SD)). He was hospitalised during the first year of life for feeding difficulties. Ichthyosis was noticed. He was able to sit at 10 months of age, and walked unsupported at 19 months, but with frequent falls. His first words appeared around 20 months of age. However, the patient presented behavioural disturbance, walking difficulties, and loss-of-consciousness spells. At 3 years of age, clinical examination revealed mild facial dysmorphism (arched eyebrows, high arched palate) and mild ataxia associated with swallowing difficulties. MRI showed hyperintense signals in the periventricular white matter compatible with delayed myelination, and an abnormal corpus callosum. After the age of 4 years, his walking ability deteriorated, leading to frequent falls with worsening of the ataxia, and subsequent loss of walking at 7 years. Sphincter control was lost at 4.5 years. Other symptoms included hepatomegaly, hypersialorrhoea, absence seizures, and very agitated behaviour. Speech remained extremely delayed.

Patient 6 was born to first-cousin parents. He was 2 years old when psychomotor delay, generalised tremor, and ataxia were noticed. He could walk a few steps with assistance and displayed irritable behaviour. Shortly afterward, he experienced severe and rapid regression with global hypotonia and severe ataxia. Brain MRI revealed progressive cortical and cerebellar atrophy. Social contact was eventually lost (no speech and no visual contact). Morphologically, he presented with hirsutism and delayed growth with delayed bone age. At the last follow-up at 8.5 years of age, the patient was unable to sit and displayed spasticity leading to hip dislocation.

Patient 7 was the second child of first-cousin parents. He presented with neonatal ichthyosis, repeated otitis, and bronchitis since 3 months of age. He experienced hypotonia from the first month of life, was able to sit at 1 year, and could stand up with assistance at 2 years. MRI showed delayed myelination. He did not present MPS features, but exhibited progressive microcephaly. His condition rapidly deteriorated. He developed retinitis pigmentosa at 3 years without corneal clouding, followed by swallowing difficulties from 4 years of age. At 6 years of age, he was bedridden and suffered from severe spasticity. His weight was 16.5 kg (−3 SD) and his height was 98.5 cm (<3 SD).

Patient 8 was born at term to healthy non-consanguineous parents after a pregnancy marked by reduced foetal movements and oligoamnios. At birth, the only symptom was the presence of dry skin. At 2 months of age, he displayed signs of tracheomalacia and hepatosplenomegaly, followed by corneal opacities at 9 months. Psychomotor delay and severe muscular hypotonia were noted when he was 1 year old. He could walk with help and could pronounce a few simple words at 2 years. By the age of 5 years, he had lost social contact and become bedridden. Serial MRI examinations revealed delayed myelination. The skin was ichthyotic, and skeletal deformities (pectus carinatum and bowed legs) were noted. Hearing loss was found at 6 years of age. He died a few months later from respiratory complications.

Patient 9 was born at 35 weeks of gestation by caesarean section following foetal cardiac arrhythmia. Hygroma, intrauterine growth retardation, and oligoamnios were noticed during pregnancy. At birth, the patient suffered from severe respiratory distress caused by choanal atresia. The first months of life were marked by severe growth retardation and repeated upper airway infections. Psychomotor delay was noted at the age of 4 months. However, at 1 year of age, the patient was able to sit, stand, and crawl. Ophthalmological examination revealed bilateral cataracts and retinal atrophy. Skeletal X-rays revealed vertebral anomalies. Ichthyosis and hepatosplenomegaly were also noticed. The child retained good social contact with her environment. MRI performed at 17 months showed mild hyperintensities in the frontal lobe white matter. Motor and mental regression occurred from the age of 2 years. Swallowing difficulties led to malnutrition and subsequent enteral feeding from the age of 2 years and 9 months. Communicating hydrocephalus was diagnosed at 3 years and 3 months, and associated with vomiting, drowsiness, and pyramidal signs. She died at the age of 3 years and 9 months.

Patient 10 was the second child of non-consanguineous parents. During the pregnancy, nuchal translucency was noticed at 9 weeks, followed by intrauterine growth retardation. At birth, she presented respiratory distress, axial hypotonia associated with limb hypertonia, pectus excavatum, thoracolumbar kyphosis, and MPS-like dysmorphic features. In the following months, she suffered from recurrent otitis and bronchitis requiring surgical intervention, severe deafness, and severe hydrocephaly requiring ventriculo-peritoneal shunting at 26 months. Severe sleep apnoea and bilateral inguinal hernia were also present, and feeding difficulties required gastrostomy tube feeding at 2.5 years of age. She presented severe dysostosis that required bracing. She died at the age of 3 years and 4 months.

The main clinical features of the patients are summarised in Table 1. Based on the classification previously proposed by Eto and coworkers [5], which takes into account the main symptoms and their ages of onset, specific clinical subtypes (i.e., juvenile, late infantile mild, late infantile severe, neonatal severe) were attributed to the patients. The phenotype of Patient 1 was of particular interest, because only rare cases of juvenile forms of MSD have been published to date. The above classification did not perfectly correlate with the disease courses and severities, as some patients within the same group displayed faster disease progression than other patients (see Disease severity, Table 1).

**Table 1:** **Clinical presentation of MSD patients**

**Patients**	**1**	**2**	**3**	**4**	**5**	**6**	**7**	**8**	**9**	**10**
**Origin**	France	France	France	France	France	Pakistan	Turkey	Lebanon	France	France
**Consanguinity**	-	-	-	-	-	+	+	-	-	-
**Antenatal manifestations (oligoamnios, IUGR)**	-	-	-	-	-	-	-	+++	+++	+++
**Age of onset (main symptoms)**	3 years (motor delay)	Birth (hernia)	12 months (psychomotor delay)	15 months (psychomotor delay)	12 months (psychomotor delay)	26 months (tremor)	3 months (hypotonia)	Birth (ichthyosis)	Birth (growth retardation, respiratory difficulties)	Birth (hypotonia, dysmorphism, respiratory distress)
**Psychomotor retardation**	++	++	+++	++	+++	++	+++	+++	+++	+++
**Neurodegeneration Neurological symptoms**	+	++	++	++	++	++	++	+++	+++	+++
**Ichthyosis**	+	+	+	-	+++	-	+++	+++	+++	++
**Dysmorphism**	-	+	-	-	++	-	-	-	-	+++
**Organomegaly**	-	-	-	-	+	ND	-	+++	+++	++
**Skeletal changes**	-	-	-	+	-	+	-	+++	+++	+++
**Heart disease**	-	-	-	-	-	-	-	-	-	++
**Corneal clouding**	-	-	-	-	-	-	-	+++	-	-
**Retinitis pigmentosa**	+	+	-	-	-	-	+	-	-	+++
**Hydrocephalus**	-	-	-	-	-	-	-	-	-	+++
**Miscellaneous**	-	Hernia, otitis	Oculomotor apraxia, myopia, livedo	Otitis, hearing loss, feeding difficulties, retinal pallor sleep disorders	Agitated behaviour, serous otitis	Hirsutism, hip dislocation	Severe growth retardation, hearing loss, otitis, spasticity	Tracheomalacia, hearing loss, hernia	Cataract, retinal atrophy, respiratory difficulties	Severe growth retardation, tracheomalacia, hearing loss, hernia
**Ongoing psychomotor regression**	No	From 6 years	From 2.5 years	No	From 3.5 years	From 2 years	From 2 years	From 2 years	From 2 years	ND
**Age of death**	Alive at 12	Alive at 11 years 3 months	Alive at 10 years and 6 months	Alive at 6	Alive at 7	Alive at 8 years and 6 months	Alive at 6 years	Dead at 6 years	Dead at 3 years and 9 months	Dead at 3 years and 4 months
**Clinical subtype** [5]	J	LIM	LIM	LIM	LIM	LIM	LIM	LIS	LIS	NS
**Disease course**	very slow	slow	slow	slow	slow	fast	fast	fast	very fast	very fast

Disease severity was estimated as described [5]. Age at onset: +++, < 2 years; ++, 2–4 years; +, > 4 years. Disease course was appreciated as follows: very slow: only mild neurological and motor involvement at 12 years old; slow: able to walk at least up to 6 years old; fast: loss of contact and/or bedridden state occurring before 7 years old; very fast: death occurring before 4 years old. *Abbreviations: IUGR* Intrauterine growth retardation, *ND* not determined, *J* juvenile, *LIM* late infantile mild, *LIS* late infantile severe, *NS* neonatal severe.

### Biochemical data

The residual sulfatase activities measured in the patients’ leukocytes are shown in Table 2. To allow comparisons among data obtained from different laboratories, the sulfatase activities are presented as percentages of control values. No clear correlations appeared between the residual enzyme activity of a given sulfatase and the disease severity. However, when patients were classified according to the average residual activities of all tested sulfatases, this parameter tended to be inversely correlated with the disease severity. Only Patients 4 and 5 showed discordant residual sulfatase activities with respect to their disease severity. Of note, similarly elevated activities were observed for Patient 5 when controlled in immortalised lymphoblasts, and the activities were even higher in fibroblasts (data not shown).

**Table 2:** **Biochemical findings in MSD patients**

**Patients**	**1**	**2**	**3**	**4**	**5**	**6**	**7**	**8**	**9**	**10**
**Sulfatases (% of control)**										
ArsA	4	14	10	5	3	3	5	2	8	7
ArsB	16	13	6	5	ND	7	ND	9	4	ND
ArsC	15	6	ND	4	ND	2	ND	ND	7	ND
IDS	7	3	4	2	20	ND	3	1	ND	0
SGSH	ND	19	ND	ND	ND	ND	ND	0.5	ND	0
G6S	ND	0	ND	ND	ND	ND	ND	ND	ND	2
GalNS	ND	5	ND	ND	28	2	ND	ND	ND	ND
**Mean**	11	9	7	4	17	4	4	3	6	2
**Urinary GAG (mg/mmol creatinine)**	0.1; 0.2		GAG: 18.8; 14.4	GAG: 0.3	GAG: 18.1; 11	ND	GAG: 12.5	ND	GAG: 22	GAG: 33
	(control: <1.8)		(control: <13)	(control: <2.6)	(control: <13)		(control: <3.3)		(control: <8)	(control: <6.3)
	HS: 20%;	HS:+++	HS: traces				HS++++; CS+++		HS: 88.5%;	HS+++; CS+++
	CS: 80%;	DS: +++					DS+; KS: traces		CS: 11.5%; no	DS++; no KS
	no DS								DS; no KS	
**Urinary sulfatides (nmol/mmol creatinine)**	88 (control: < 40)	positive	ND	positive	263 (control: < 40)	ND	ND	ND	ND	ND

Sulfatase activities were determined on lysates of peripheral blood leukocytes, and are expressed as percentage of the values measured in control samples. Abnormal urinary excretion of glycosaminoglycans and sulfatides is indicated (note that distinct methods were employed by different laboratories). *Abbreviations: ArsA* arylsulfatase A, *ArsB* arylsulfatase B, *ArsC* arylsulfatase C, *IDS* iduronate 2-sulfatase, *SGSH* N-sulfoglucosamine sulfohydrolase, *G6S *glucosamine 6-sulfatase, *GalNS* galactosamine 6-sulfatase, *GAG *glycosaminoglycans, *HS* heparan sulfate,* DS* dermatan sulfate, *KS* keratan sulfate, *CS* chondroitin sulphate, *ND* not determined.

### Molecular analyses

Genomic DNA and/or cDNA sequencing revealed changes in the *SUMF1* sequence on both alleles of each patient. We thus identified 13 different mutations, nine of which have not previously been described (Table 3). The mutations included nonsense, missense, and splicing site mutations. It is noteworthy that a duplication of four entire exons without inducing a frameshift was identified in the cDNA of Patient 7. Sequencing of all exons and intron–exon junctions in the genomic DNA failed to reveal any anomaly. Similarly, the skipping of exon 3 and exon 5 in the cDNA of Patient 5 and Patient 10, respectively, did not lead to a frameshift. However, the use of specific primers revealed that the mutation leading to exon 3 skipping in Patient 5 generated another transcript retaining the first four nucleotides of intron 3 and lacking the first seven nucleotides of exon 4. This splicing could be predicted by Human Splicing Finder software [22]. Unexpectedly, this change in the genomic sequence induced the deletion of only one alanine residue at position 175.

**Table 3:** **SUMF1 mutations identified in MSD patients**

**Patient**	**Mutation**	**Affected Exon**	**Mutant protein**	**Predicted effect of missense mutations on the FGE structure**
**1**	c.[463 T > C] + **[1029G > T]**	3 and 9	p.[S155P] + **[R343S]**	p.S155P: loss of three hydrogen bonds and steric clash with Ala186 [12]. p.R343S: direct interaction with both sulfhydryl groups of the catalytic Cys336/Cys341 pair. Might affect substrate binding and reactivity.
**2**	c.**[**836C > T] + **[**836C > T]	6	p.[A279V] + [A279V]	Steric clash with Phe275 and Ala283 [12].
**3**	c.**[**836C > T] + **[**836C > T]	6	p.[A279V] + [A279V]	Idem
**4**	c.**[**836C > T] + **[**836C > T]	6	p.[A279V] + [A279V]	Idem
**5**	**c.[893C > A]** + **[519 + 4A > G]**	7 and 3	**p.[A298E]** + **[A149_A173del, A175del]**	p.A298E: neither affects FGE fold nor binding to the Ca^2+^ ion at site 2.
**6**	c.**[**788G > T**] + [**788G > T**]**	6	p.[G263V] + [G263V]	Steric clash with Thr263 and Thr270 [12].
**7**	**c.[520_954dup (+) 520_954dup]**	4 to 7	**p.[V174_P318dup] + [V174_P318dup]**	
**8**	c.**[706C > T]** + [1045C > T]	5 and 9	**p.[R236X]** + **[**R349W]	p.R349W: loss of five interactions [12]. Should affect protein fold in the vicinity of the active site.
**9**	c.**[132_133insG]** + [1045C > T]	1 and 9	**p.[V45fsX75] + [**R349W]	idem
**10**	**c.[776A > G;1018 T > C]** + **[725 + 1G > C]**	6, 9 and 5	**p.[N259S; Y340H]** + **[P202_R242del]**	p.N259S precludes binding to Ca^2+^ ion at site 1. p.Y340H should not induce major structural changes.

Novel mutations are indicated in bold characters. Patients are listed as in Table 1, where they are ranked on an ascending scale of disease severity*.*

**Figure 1:**
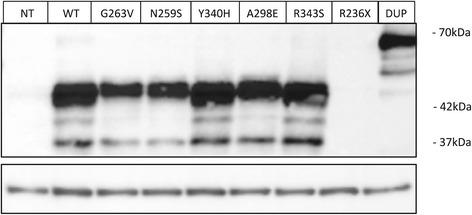
**Western blot analysis of overexpressed wild-type and mutant FGE.** Lysates of HEK293T cells transiently transfected with a vector encoding C-terminally flagged wt or mutant FGE (25 μg protein) were assessed for FGE expression by Western-blot. Upper panel: FGE. Lower panel: loading control. NT, not transfected; DUP, p.V174-P318dup.

To further characterise the impacts of the *SUMF1* mutations on the expression of the protein, we individually expressed some of the mutants identified. Protein expression and stability were evaluated by western blotting analysis after transient overexpression of the C-terminally Flagged mutants in HEK293T cells (Figure 1). The anti-Flag antibody revealed three bands, comprising an approximately 42-kDa band corresponding to full-length FGE and two bands corresponding to N-terminally processed forms intended for secretion, as previously reported [27]. While the p.Y340H and p.R343S mutants were expressed at the same level as the wild-type (wt) protein, the p.G263V and p.N259S mutants showed reduced expression. The p.A298E mutant was expressed at an intermediate level. As expected, the mutant carrying the p.R236X nonsense mutation could not be detected, because the C-terminal Flag-tag was not translated in this construct. The p.V174-P318dup mutant carrying the duplication of four exons was efficiently expressed and appeared at around 65 kDa.

We aimed to evaluate the FGE function based on the ability of each mutant to restore sulfatase activities in a defective MSD fibroblast cell line. For this purpose, the mutants were stably overexpressed in MSD fibroblasts, and the arylsulfatase A, B, and C activities were measured. Unfortunately, the expression levels of FGE in the stable transfectants were not sufficiently high to allow accurate assessment of the protein functionality. Of note, however, and despite several attempts, no stable cell transfectants could be obtained for the p.V174-P318dup mutant, suggesting that it may exert some cytotoxicity.

**Figure 2:**
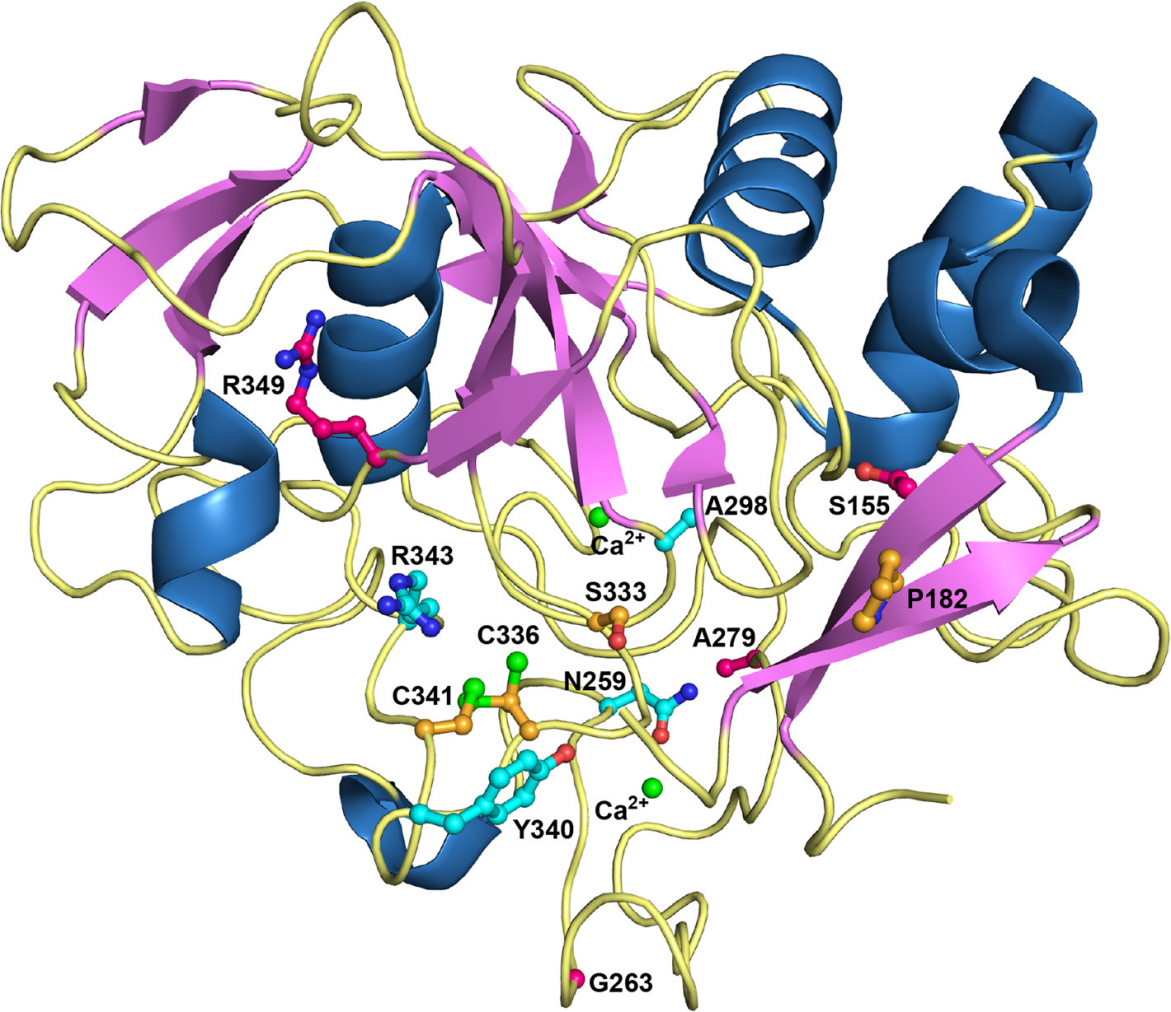
**Representation of FGE and the main mutated residues identified in this and previous studies.** Cartoon representation of the FGE structure (PDB code 1Y1E) with helices in blue, beta-strands in violet, and loops in yellow. Represented in ball-and-stick and labelled are (i) essential or important residues for substrate binding and catalytic activity (orange carbon atoms), (ii) residues whose mutations have been previously described (magenta carbon atoms) [6,16,17,27], and (iii) newly identified mutations (cyan carbon atoms). N, O, and S atoms are in blue, red, and green, respectively. The two structural calcium ions are also represented (green spheres).

**Figure 3:**
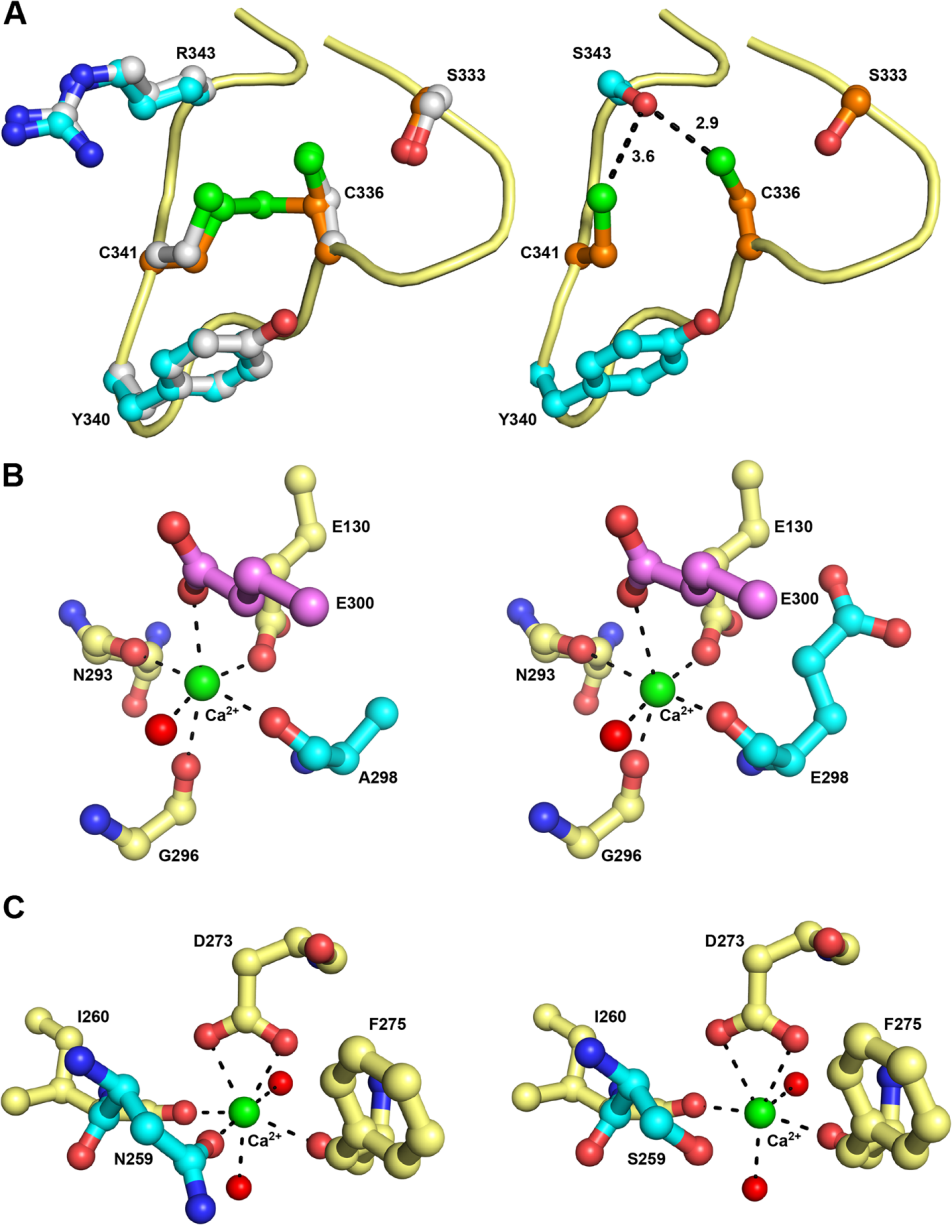
**Structural modifications of FGE due to mutations identified in MSD patients.** Details of the conformation of wt (left panel) and mutant (right panel) FGE are shown for three missense mutations: p.R343S **(A)**; p.A298E **(B)** and p.N259S **(C)**.

Structural analyses were performed to complete the characterisation of the missense *SUMF1* mutations, based on the known 3D structures of FGE [12,13] (Figures 2 and 3).

The structural consequences of the p.S155P, p.G263V, p.A279V, and p.R349W mutations have already been reported [12], and will not be discussed here. Besides the previously described p.S155P mutation, Patient 1 also carried the novel p.R343S substitution. Arg343 is strictly conserved within all species, and the p.R343S mutation is “probably damaging” (with the highest score) according to the Polyphen prediction software. Interestingly, the Arg343 side-chain, which adopts two discrete conformations in the wt enzyme structure, points toward and establishes a long-range interaction with the sulfhydryl group of Cys341 when the catalytic Cys336/Cys341 pair is reduced (Figure 3A, left). The substitution of Arg343 to a serine, wherein the molecular modelling revealed that the hydroxyl group may directly interact with the sulfhydryl groups of both Cys341 and the more reactive Cys336 (dS343OG-C341SG = 3.6 Å; dS343OG-C336SG = 2.9 Å) (Figure 3A, right) [14], might have important consequences in terms of substrate binding and reactivity. Among the other newly identified mutations, Patient 5 carried a p.A298E substitution. Ala298 is well-conserved among animal species, but sometimes replaced by a valine. The Polyphen software predicted that this mutation is “possibly damaging” with a score of 0.893. In the structure of wt FGE, the carbonyl group of A298 is involved in the perfect octahedral coordination of the Ca^2+^ ion at site 2 (Figure 3B, left). Molecular modelling of p.A298E indicates that a glutamate side-chain could easily be accommodated by the FGE fold and that the main-chain carbonyl group would point in the same direction as in the wt protein, and probably not affect the Ca^2+^ ion coordination sphere (Figure 3B, right). Finally, with respect to Patient 10, Asn259 was previously proposed to be a critical residue for the FGE protein (see Discussion). In the p.N259S mutant identified in Patient 10, the overly-long distance between the oxygen atom of the serine hydroxyl group and the calcium ion (3.5 Å) would preclude the establishment of a coordinate bond (Figure 3C, right). The Tyr340 residue, which is also mutated to histidine in Patient 10, is very close to the catalytic site of FGE. Nevertheless, the substitution of the tyrosine by a histidine does not induce any steric hindrance, because the aromatic and imidazole groups are approximately the same size.

## Discussion

The clinical features and gene defects of 10 new patients affected with the rare disorder MSD were analysed. Through detailed clinical descriptions of each patient, together with biochemical and molecular characterisations, we investigated the possible predictive values of some of the identified features for disease progression and outcome.

### Clinical heterogeneity, correlation between biochemical and clinical phenotypes

In the search for prognostic factors, we first determined the extent of disease severity for each patient. The disease course, assessed through the clinical history and age of death (when available), appeared to be a relatively good marker of disease severity (see Table 1). Meanwhile, the clinical subtypes (i.e., juvenile, late infantile mild, late infantile severe, neonatal severe) were distinguished according to the classification proposed by Eto and coworkers [5], which takes into account the main symptoms and their ages of onset. The subtypes obtained with this historical classification were not perfectly correlated with the disease courses. Indeed, some patients within the same clinical subtype displayed more rapid disease progression than other patients (see Table 1). We thus chose to define the disease severity according to the disease course and then aimed to determine the clinical features that could influence the prognosis.

The age at onset of the first symptoms appeared to be highly variable and did not correlate well with the disease course and severity (e.g., in Patient 2). Psychomotor retardation and hypotonia were common to all patients, around the age of 2 years. While these symptoms appeared at an earlier time in the most severe forms (before 1 year of age for Patients 7, 8, 9, and 10), this correlation did not hold true for all patients (e.g. Patient 3 with psychomotor delay noticed as early as 1 year of age with slow disease evolution). The onset of psychomotor regression appeared to be a more accurate indicator (see “ongoing psychomotor regression”, Table 1). When observed at 2 years of age, it was associated with rapid disease progression, whereas later onset was linked to a slower disease course. Finally, the presence of several non-neurological symptoms (e.g., dysmorphism, skeletal abnormalities, organomegaly) before 2 years of age predicted a more severe and rapid form of the disease (see Table 1, Patients 8, 9, and 10). Taken together, these observations identify two major clinical features that could be in favour of rapid progression of the disease, namely the presence of several non-neurological symptoms and the onset of psychomotor regression before 2 years of age.

We also examined the possibility of stratifying patients based on the mean residual sulfatase activities. Our observations indicated the presence of a trend for the residual sulfatase activities in leukocytes to be inversely correlated with the disease severity. However, this conclusion, which corroborates a previous statement [5], can only be drawn in a global context of comparison. It would be illusive to give a prognosis based solely on the measurement of sulfatase activities, because the results are clearly dependent on the nature of the biological samples examined [28], the enzyme assay protocol, and the sulfatase under analysis [16]. In addition, our results confirmed previous observations indicating that sulfatases are heterogeneously affected by *SUMF1* defects [16]. Analyses of additional MSD cases are warranted to investigate whether certain sulfatases are preferentially affected by the identified *SUMF1* mutations, or whether the heterogeneous impacts arise through epigenetic or environmental factors.

### Molecular analysis and genotype-phenotype correlations

More than 30 *SUMF1* mutations have been reported to date. The impacts of many of these mutations on the FGE structure, subcellular localisation, and activity have been characterised (see Additional file 2). Hence, several attempts to establish genotype–phenotype correlations have been made, outlining some concepts. The neonatal severe form of the disease may be caused by a combination of nonsense or missense mutations directly affecting the FGE catalytic site [18]. Missense mutations with severe impacts on either the protein stability or catalytic activity would lead to severe phenotypes, whereas those only partially affecting the protein stability would be associated with milder phenotypes [27]. Finally, the human mutations may all be hypomorphic, although this statement has been challenged by a severe neonatal case associated with nonsense mutations in both alleles [27].

The analyses of the 10 MSD patients reported here allowed further examination of genotype–phenotype correlations. Among the 13 *SUMF1* molecular defects identified, nine were novel mutations, comprising four missense mutations, two nonsense mutations, one duplication, and two splice site mutations. The most striking observation related to Patient 1. This patient carried the previously described p.S155P mutation. Ser155 lines the substrate-binding groove. Four homoallelic p.S155P cases have been reported to date [16,27], and all four patients had severe disease presentation. Although discordant results were obtained *in vitro*, this mutation clearly affected the FGE expression and activity (2% of wt) [27]. In Patient 1, the other allele carried the newly identified p.R343S substitution. Our results suggested that this substitution may impact the FGE catalytic activity. Thus, there is a discrepancy between the clearly attenuated phenotype observed in this patient and his genotype. Once more, these observations underscore the real difficulty in establishing rules about genotype–phenotype correlations.

The p.A279V mutation has already been reported elsewhere [17]. Together with Patients 2, 3, and 4, there are now four reported French patients homozygous for this mutation despite being born to non-consanguineous parents. Therefore, the p.A279V mutation is likely to be relatively common in the French population. Moreover, all four patients presented a late infantile mild phenotype. In addition, our three patients exhibited similar, relatively slow deterioration. These observations underline a genotype–phenotype correlation for this mutation.

Patient 5 carried the p.A298E substitution. In our hands, this substitution by glutamic acid appeared to have little impact on the protein expression and conformation (Figures 1 and 3B). On the other allele, the patient carried a mutation in the donor site of intron 3, leading to exon 3 skipping. Skipping of exon 3 has already been reported along with nonsense mutations in at least two patients, leading to neonatal severe phenotypes [6,7,27]. It is thus consistent that, when this mutation was associated with a missense mutation, such as in Patient 5, it led to a milder phenotype. A third transcript was identified in Patient 5, which induced deletion of Ala175 at the protein level. Residue 175 is located in the Ser163-Ala176 segment that is absent from the FGE protein structure as a result of proteolysis by elastase treatment [12]. This segment probably corresponds to an exposed loop at the surface of the FGE structure and the impacts of the deletion on the stability and activity of the corresponding variant cannot easily be predicted. In addition, the contribution of this mutant to the phenotype of Patient 5 is difficult to assess because it is produced from a transcript that is likely to be quantitatively minor.

Two cases of p.G263V homozygosity, associated with late infantile mild phenotypes, were recently reported [27]. Structural and molecular analyses revealed that p.G263V has a greater impact on the FGE protein stability (confirmed by our expression analysis, Figure 1) than on its catalytic activity, which averaged 15% of the wt control [27]. These observations lead to the conclusion that the p.G263V mutation induces a relatively attenuated form of the disease. We report here a novel patient homozygous for the p.G263V mutation (Patient 6). His presentation, which was mainly neurological, was also reminiscent of the late infantile mild subtype. However, the patient’s condition worsened relatively quickly. Thus, homozygosity for the p.G263V mutation does not preclude a severe prognosis.

Patient 7 was homozygous for a mutation leading to a large duplication encompassing exons 4 to 7, without inducing a frameshift. Such a large duplication in the *SUMF1* gene has not previously been described. Surprisingly, while this duplication mutant could be transiently expressed in HEK293T cells, several attempts to obtain stable transfectants in fibroblasts were unsuccessful. The issue of whether the potential toxicity of this mutant could account for the severe phenotype of this patient remains to be investigated.

Patient 8 carried a nonsense mutation on one allele, together with the well-characterised p.R349W substitution on the other allele. The p.R349W mutation has already been reported in the Turkish population [17]. It was shown to dramatically affect both the FGE stability and catalytic activity, and was always associated, in an homozygous context, with severe phenotypes [6,7,16,17]. The p.R236X mutation found in the other allele has not previously been described. It induces the loss of 139 amino acids, including critical residues for the protein stability and enzyme activity. Such a large modification may prevent the protein from passing the endoplasmic reticulum quality control system. In addition, the possibility that the mRNA carrying a premature termination codon is recognised and processed by mRNA decay cannot be ruled out. As expected, the association of the p.R349W and p.R236X mutations led to a severe phenotype that we qualified as an infantile severe form. Of note, the sulfatase activities measured in the patient’s leukocytes were very low.

A comparable genotype was found in Patient 9. The p.R349W mutation was associated with a novel nonsense mutation, p.V45fsX75. The same conclusions as those drawn for Patient 8 regarding genotype–phenotype correlations could be expected. Indeed, as shown in Table 1, the phenotypes of Patients 8 and 9 were strikingly similar, including intrauterine manifestations, early presentation of non-neurological symptoms, and rapid neurologic regression. In addition, these two patients shared the same non-neurological symptoms. Finally, both patients died at an early age.

Patient 10 proved to have a complex genotype. In addition to several polymorphisms (data not shown), she carried two missense mutations on one allele (paternally inherited) and a splice site mutation on the other allele, leading to exon 5 skipping. Skipping of exon 5 (albeit through another genomic mutation) has already been reported once in heterozygosity with the p.A279V mutation, leading to a “moderate” phenotype [6]. Exon 5 skipping induces the loss of two disulphide bonds that appear to be essential for protein folding, and is therefore likely to have a profound impact on the enzyme function. The other allele carried two missense mutations, p.Y340H and p.N259S, which have not previously been reported. Overexpression of each mutant individually was of great interest in that case, because it allowed us to identify the disease-causing mutation. The p.Y340H mutation had little impact on the protein stability, a finding in agreement with our structural analysis, which did not reveal any major modifications. Besides, a p.Y340F mutant was previously reported to conserve 74% of the wt enzyme activity [12]. Taken together, these data suggest that the Y340 substitution had little impact on FGE. However, the p.N259S mutation appeared to have much more severe consequences for the FGE protein. We demonstrated that this mutation impaired FGE expression (Figure 1). The Asn259 residue is thought to play a critical role in FGE stability, by participating in the coordination of one of the two structurally important calcium ions [12] (see also Figure 3C, right). In addition, a p.N259I mutant was previously shown to be totally unable to activate arylsulfatases A, C, and E [16]. Finally, the p.N259S mutation appeared to largely contribute, in association with exon 5 skipping, to the severe phenotype of Patient 10.

## Conclusions

This study provides new insights into MSD, a disorder that remains poorly known. Special attention was paid to highlighting signs and factors that could serve as prognostic indicators. Based on clinical observations, the presence of non-neurological symptoms before the age of 2 years and the occurrence of psychomotor regression around the same age appear to be associated with rapid progression of the disease. Meanwhile, a correlation between the mean residual leukocyte sulfatase activities and the clinical severity was observed, although the activities of individual sulfatases were not reliable prognostic markers. These findings are in line with previously proposed statements regarding genotype–phenotype correlations. The present findings confirm the involvement of null mutations (e.g., p.R236X and p.V45fs75X) or mutations that deeply modify the FGE sequence (exon duplication or skipping) in severe phenotypes. Our results also demonstrate the severe impact of the p.R349W mutation. Missense mutations that affect the protein expression (i.e., p.N259S and p.G263V) appear to be clearly deleterious. However, missense mutations that moderately affect the FGE stability and are not thought to directly target the catalytic site (i.e., p.A279V and p.A298E) seem to be associated with relatively milder phenotypes and slower disease progression. Nevertheless, discrepancies were noted for the p.R343S mutant, which was predicted by molecular analysis to be deeply damaging for the FGE activity but was associated with the mildest phenotype in this study. Full descriptions of additional MSD patients should help to clarify the pathogenicity of this and other mutants.

## Additional files

Additional file 1:
**Primers for site-directed mutagenesis.** The expression plasmids generated encode C-terminally flagged FGE mutants. Additional file 2:
**Characteristics of SUMF1 mutations.** The consequences of SUMF1 mutations on FGE (including FGE subcellular localization, sulfatase and FGE activity) and the patient’s clinical phenotype are indicated. Data on FGE derive from individually expressed SUMF1 mutations in cellular systems. Note that mutations that have not been characterized by the above criteria are not included. Abbreviations: ArsA, arylsulfatase A; ArsB, arylsulfatase B; ArsC, arylsulfatase C; IDS, iduronate 2-sulfatase; SGSH, N-sulfoglucosamine sulfohydrolase; ER: endoplasmic reticulum; LIM: late infantile mild; LIS: late infantile severe.
