# Supplementary material for: Natural disease history and characterisation of SUMF1 molecular defects in ten unrelated patients with multiple sulfatase deficiency
Source: Orphanet J Rare Dis. 2015 Mar 15;10:31. doi: 10.1186/s13023-015-0244-7 (PMC4375846; doi:10.1186/s13023-015-0244-7)
Supplement: Additional file 1: — Primers for site-directed mutagenesis. The expression plasmids generated encode C-terminally flagged FGE mutants. [file 13023_2015_244_MOESM1_ESM.docx]

| Vector | Primers |
| --- | --- |
| pR236X-Flag | Forward: ^5’^GGGAATACAGCTGTTGAGGAGGCCTGC^3’^  Reverse: ^5’^GCAGGCCTCCTCAACAGCTGTATTCCC^3’^ |
| pN259S-Flag | Forward: ^5’^GGCCAGCATTATGCCAGCATTTGGCAGGGCG^3’^  Reverse: ^5’^CGCCCTGCCAAATGCTGGCATAATGCTGGCC^3’^ |
| pG263V-Flag | Forward: ^5’^GCCAACATTTGGCAGGTCGAGTTTCCGG^3’^  Reverse: ^5’^CCGGAAACTCGACCTGCCAAATGTTGGC^3’^ |
| pA298E-Flag | Forward: ^5’^ CAACATAGTGGGGAACGAATGGGAATGGACTTCAGAC^3’^  Reverse: ^5’^ GTCTGAAGTCCATTCCCATTCGTTCCCCACTATGTTG^3’^ |
| pY340H-Flag | Forward: ^5’^CCTACATGTGCCATAGGTCTCATTGTTACAGGTATCGC^3’^  Reverse: ^5’^GCGATACCTGTAACAATGAGACCTATGGCACATGTAGG^3’^ |
| pR343S-Flag | Forward: ^5’^ GGTCTTATTGTTACAGTTATCGCTGTGCTGCTCG^3’^  Reverse: ^5’^ CGAGCAGCACAGCGATAACTGTAACAATAAGACC^3’^ |

### Additional file 1 – Primers for site-directed mutagenesis.

### The expression plasmids generated encode C-terminally flagged FGE mutants
