# Supplementary material for: Natural disease history and characterisation of SUMF1 molecular defects in ten unrelated patients with multiple sulfatase deficiency
Source: Orphanet J Rare Dis. 2015 Mar 15;10:31. doi: 10.1186/s13023-015-0244-7 (PMC4375846; doi:10.1186/s13023-015-0244-7)
Supplement: Additional file 2: — Characteristics of SUMF1 mutations. The consequences of SUMF1 mutations on FGE (including FGE subcellular localization, sulfatase and FGE activity) and the patient’s clinical phenotype are indicated. Data on FGE derive from individually expressed SUMF1 mutations in cellular systems. Note that mutations that have not been characterized by the above criteria are not included. Abbreviations: ArsA, arylsulfatase A; ArsB, arylsulfatase B; ArsC, arylsulfatase C; IDS, iduronate 2-sulfatase; SGSH, N-sulfoglucosamine sulfohydrolase; ER: endoplasmic reticulum; LIM: late infantile mild; LIS: late infantile severe. [file 13023_2015_244_MOESM2_ESM.docx]

### Additional file 2 – Characteristics of *SUMF1* mutations.

### The consequences of *SUMF1* mutations on FGE (including FGE subcellular localization, sulfatase and FGE activity) and the patient’s clinical phenotype are indicated. Data on FGE derive from individually expressed *SUMF1* mutations in cellular systems. Note that mutations that have not been characterized by the above criteria are not included. Abbreviations: ArsA, arylsulfatase A; ArsB, arylsulfatase B; ArsC, arylsulfatase C; IDS, iduronate 2-sulfatase; SGSH, N-sulfoglucosamine sulfohydrolase; ER: endoplasmic reticulum; LIM: late infantile mild; LIS: late infantile severe.

| **Mutations** | **Locali-zation** | **Intracellular expression level** | **Sulfatase activities**  **(% of wt)** | **FGE activity**  **(% of wt)** | **Patient’s phenotype** |
| --- | --- | --- | --- | --- | --- |
| p.L20F |  |  | 42 (ArsA) ; 44 (ArsC) ; 10 (ArsE) [16] |  |  |
| p.E130D | ER [29] | Severely reduced [29] |  | 55 [29] |  |
| p.S155P | ER [15, 27] | Not reduced [15]; severely reduced [27] | 22 (ArsA) ; 25 (ArsC) ; 7 (ArsE) [16]  17 (ArsA) ; 24 (ArsC) ; 21 (IDS) ;41 (SGSH) [15] | 2 [27] | p.[S155P]+[S155P]  Severe [16]; “Moderate” [6]; LIS [27] |
| p.A177P | ER [17] | Not reduced [17] | 5 (ArsA) ; 22 (ArsC) ; 2 (ArsE) [16] | 0.6 [17] |  |
| p.W179S | ER [17] | Slightly reduced [17] |  | 2.7 [17] | p.[W179S]+[W179S]  LIM [17] |
| p.C218Y |  |  | 3 (ArsA) ; 11 (ArsC) ; 8 (ArsE) [16] |  |  |
| p.R224W | ER [15] | Not reduced [15] | 2 (ArsA) ; 5 (ArsC) ; 24 (Ars E) [16]  18 (ArsA) ; 21 (ArsC) ; 23 (IDS) ;32 (SGSH) [15] |  | p.[R224W]+[R224W]  Mild [16] |
| p.G247R | ER [27] | Significantly reduced [27] |  | 7 [27] | p.[G247R]+[G247R]  LIS [27, 30, 31] |
| p.G263V | ER [27] | Significantly reduced [27] |  | 16 [27] | p.[G263V]+[G263V]  LIS [27] |
| p.N259I |  |  | 1 (ArsA) ; 0,5 (ArsC) ; 1 (ArsE) [16] |  |  |
| p.P266L |  |  | 55 (ArsA) ; 81 (ArsC) ; 98 (ArsE) [16] |  |  |
| p.A279V | ER [17] | Severely reduced [17] | 15 (ArsA) ; 43 (ArsC) ; 41 (ArsE) [16] | 22.9 [17] | p.[A279V]+[A279V]  LIM [17] |
| p.R327X | ER [27] | Severely reduced [27] |  | 0 [27] |  |
| p.S333A |  |  |  | 5.2 [12] |  |
| p.S333T |  |  |  | 4.5 [12] |  |
| p.C336R | ER [15] | Not reduced [15] | 7 (ArsA) ; 13 (ArsC) ; 32 (ArsE) [16]  2 (ArsA) ; 2 (ArsC) ; 1 (IDS) ;1 (SGSH)[15] |  |  |
| p.H337A |  |  |  | 18.3 [12] |  |
| p.Y340F |  |  |  | 74.2 [12] |  |
| p.R345C | ER [15, 27] | Not reduced [15]  Slightly reduced [27] | 37 (ArsA) ; 95 (ArsC) ; 91 (ArsE) [16]  19 (ArsA) ; 21 (ArsC) ; 12 (IDS) ;40 (SGSH) [15] | 2 [27] | p.[R345C]+[R345C] “Mild-moderate” [6]; Severe [19]; LIS [27] |
| p.A348P |  |  | 15 (ArsA) ; 46 (ArsC) ; 32 (ArsE) [16] |  |  |
| p.R349W | ER [15, 17] | Severely reduced [17];  Not affected [15] | 28 (ArsA) ; 38 (ArsC) ; 26 (ArsE) [16]  32 (ArsA) ; 35 (ArsC) ; 28 (IDS) ;48 (SGSH) [15] | 0.5 [17] | p.[R349W]+[R349W]  Severe neonatal [6] , LIS [17] |
| p.R349Q |  |  | 22 (ArsA) ; 48 (ArsC) ; 7 (ArsE) [16] |  |  |
| p.A149_A173del (Exon 3 skipping) | ER [27] | Significantly reduced [27] | 17 (ArsA) ; 4 (ArsB) ; 35 (ArsC) [7]  35-26(ArsA); 63-67(ArsB) ; 47-16.5(ArsC) [6] | 0.3 [27] |  |
| c.[954+5G>T]+[954+5G>T] |  |  |  |  | Severe [19] |
| p.[A149_A173del]+[R327X] |  |  |  |  | Neonatal severe [6, 27] |
| p.[A149_A173del]+[S359X] |  |  |  |  | Neonatal severe [7] |
| p.[M1R]+[I93fsX108] |  |  |  |  | “Moderate” [6] |
| p.[M1V]+[A348P] |  |  |  |  | “Mild-moderate” [6] |
| p.[A177P]+[L250fs] |  |  |  |  | LIM [17] |
| p.[C218Y]+[R345C] |  |  |  |  | “Moderate” [6] |
| p.[W220fsX266]+[?] |  |  |  |  | Neonatal severe [6, 7] |
| p.[A279V]+[P202_R242del] (Exon 5 skipping) |  |  |  |  | “Moderate” [6] |
| p.[A279V]+[P81fsX139] |  |  |  |  | “common form of MSD” [7] |
| p.[R349G]+[F244S] |  |  |  |  | LIS [32] |
| p.[R349Q]+[C336R] |  |  |  |  | Severe [6] |
| p.[E130D]+[C52fsX57] |  |  |  |  | LIS [29] |

### Additional references:

29. Schlotawa L, Radhakrishnan K, Baumgartner M, Schmid R, Schmidt B, Dierks T, Gartner J: **Rapid degradation of an active formylglycine generating enzyme variant leads to a late infantile severe form of multiple sulfatase deficiency.** *Eur J Hum Genet* 2013. **21**:1020-3.

30. Yis U, Pepe S, Kurul SH, Ballabio A, Cosma MP, Dirik E: **Multiple sulfatase deficiency in a Turkish family resulting from a novel mutation.** *Brain Dev* 2008, **30:**374-377.

31. Incecik F, Ozbek MN, Gungor S, Pepe S, Herguner OM, Mungan NO, Gungor S, Altunbasak S: **Multiple sulfatase deficiency: A case series of four children.** *Ann Indian Acad Neurol* 2013, **16:**720-722.

32. Artigalas OA, da Silva LR, Burin M, Pastores GM, Zeng B, Macedo N, Schwartz IV: **Multiple sulfatase deficiency: clinical report and description of two novel mutations in a Brazilian patient.** *Metab Brain Dis* 2009, **24:**493-500.
